# Nucleotide-Binding Oligomerization Domain 1 (NOD1) Positively Regulates Neuroinflammation during Japanese Encephalitis Virus Infection

**DOI:** 10.1128/spectrum.02583-21

**Published:** 2022-05-31

**Authors:** Zheng Chen, Zikai Zhao, Yixin Liu, Muhammad Imran, Jing Rao, Ning Cai, Jing Ye, Shengbo Cao

**Affiliations:** a Department of Preventive Veterinary Medicine, College of Animal Science and Technology, Jiangxi Agricultural University, Nanchang, Jiangxi, People’s Republic of China; b State Key Laboratory of Agricultural Microbiology, Huazhong Agricultural University, Wuhan, Hubei, People’s Republic of China; c Key Laboratory of Preventive Veterinary Medicine in Hubei Province, College of Veterinary Medicine, Huazhong Agricultural University, Wuhan, Hubei, People’s Republic of China; d The Cooperative Innovation Center for Sustainable Pig Production, Huazhong Agricultural University, Wuhan, Hubei, People’s Republic of China; Wuhan Institute of Virology

**Keywords:** JEV, NOD1, NF-κB, neuroinflammation

## Abstract

Japanese encephalitis virus (JEV) is a neurotropic flavivirus that invades the central nervous system and causes neuroinflammation and extensive neuronal cell death. Nucleotide-binding oligomerization domain 1 (NOD1) is a type of pattern recognition receptor that plays a regulatory role in both bacterial and nonbacterial infections. However, the role of NOD1 in JEV-induced neuroinflammation remains undisclosed. In this study, we evaluated the effect of NOD1 activation on the progression of JEV-induced neuroinflammation using a human astrocytic cell line and NOD1 knockout mice. The results showed that JEV infection upregulated the mRNA and protein expression of NOD1, ultimately leading to an enhanced neuroinflammatory response *in vivo* and *in vitro*. Inhibition of NOD1 in cultured cells or mice significantly abrogated the inflammatory response triggered by JEV infection. Moreover, compared to the wild-type mice, the NOD1 knockout mice showed resistance to JEV infection. Mechanistically, the NOD1-mediated neuroinflammatory response was found to be associated with increased expression or activation/phosphorylation of downstream receptor-interacting protein 2 (RIPK2), mitogen-activated protein kinase (MAPK), extracellular signal-regulated kinase (ERK), Jun N-terminal protein kinase (JNK), and NF-κB signaling molecules. Thus, NOD1 targeting could be a therapeutic approach to treat Japanese encephalitis.

**IMPORTANCE** Neuroinflammation is the main pathological manifestation of Japanese encephalitis (JE) and the most important factor leading to morbidity and death in humans and animals infected by JEV. An in-depth understanding of the basic mechanisms of neuroinflammation will contribute to research on JE treatment. This study proved that JEV infection can activate the NOD1-RIPK2 signal cascade to induce neuroinflammation through the proven downstream MAPK, ERK, JNK, and NF-κB signal pathway. Thus, our study unveiled NOD1 as a potential target for therapeutic intervention for JE.

## INTRODUCTION

Japanese encephalitis virus (JEV) is a positive-sense, single-stranded RNA virus, belonging to the genus *Flavivirus* of the family *Flaviviridae*, which can cause Japanese encephalitis (JE) (1). JE is an acute and severe disease of the central nervous system (CNS), one of the foremost causes of epidemic encephalitis in humans, especially in the regions of South and Southeast Asia, eastern Russia, a few parts of Australia, and the Western Pacific islands (Saipan and Papua New Guinea) (2, 3). Severe JE gives rise to numerous physiopathological alterations, which are characterized by high fever, headache, stiffness of neck muscles, disorientation, seizures, paralysis, coma, and eventually death (4). According to a World Health Organization report, about 60% of the world’s population (nearly 3 billion) live in areas where JE is endemic, with an estimated 13,600 to 20,400 deaths annually, indicating a huge threat to human health (5).

JEV targets the CNS, causing devastating and fatal neuroinflammation characterized by neuronal destruction, microgliosis, astrogliosis, and the production of various inflammatory cytokines (6–9). Neuroinflammation is the main pathological manifestation of JE and the most important factor leading to morbidity and death in humans and animals. Several mechanisms of JEV-induced neuroinflammation have been elucidated in the past few years. The current understanding is that JEV reaches the CNS by crossing the blood-brain barrier (10–12), then infects microglia, astrocytes, and neurons, causing neuroinflammation by stimulating cell surface Toll-like receptors, platelet-derived growth factor receptors, C-C motif chemokine receptor 2, and/or cytosolic retinoic acid-inducible gene I (RIG-I) pattern recognition receptor (PRRs)-induced immune signaling cascades (13–16). Whether other PRRs can recognize JEV and activate an inflammatory response remains to be uncovered.

Nucleotide-binding oligomerization domain 1 (NOD1) belongs to the NOD-like receptor (NLR) family of PRRs, which are localized in the cytoplasm (17, 18). NOD1 has been mainly reported to sense bacterial pathogens by detecting a conserved structure of peptidoglycan in the bacterial cell wall (17). Upon sensing peptidoglycan, NOD1 signals via a caspase-activated recruitment domain and subsequently interacts with its adaptor protein, receptor-interacting protein 2 (RIPK2) (19–21). The NOD1-RIPK2 interaction results in the activation of nuclear factor kappa B (NF-κB) and mitogen-activated protein kinase (MAPK) pathways to drive inflammatory cytokine production (19, 20). A number of recent studies have also suggested a role of NOD1 in nonbacterial infections. For instance, Vegna et al. (22) found that NOD1 can be activated by the polymerase of the hepatitis C virus (HCV). Specifically, NOD1 interacts with the viral double-stranded RNA (dsRNA) to regulate the innate immune response and inflammation triggered by HCV. Although the functions of NOD1 are known in both bacterial and nonbacterial infections, its role in JEV-induced neuroinflammation is undetermined.

In this study, we found that JEV infection upregulated the expression of NOD1 in human glial cells and mouse brain tissues. Inhibition of NOD1 expression ameliorated JEV-induced neuroinflammation via dampening the NF-κB and MAPK signaling pathways both *in vitro* and *in vivo*. These data revealed a novel mechanism of JEV neuroinvasion and suggest NOD1 as a therapeutic target for treating JE.

## RESULTS

### JEV infection upregulates NOD1 and RIPK2 expression *in vitro* and *in vivo*.

**FIG 1:**
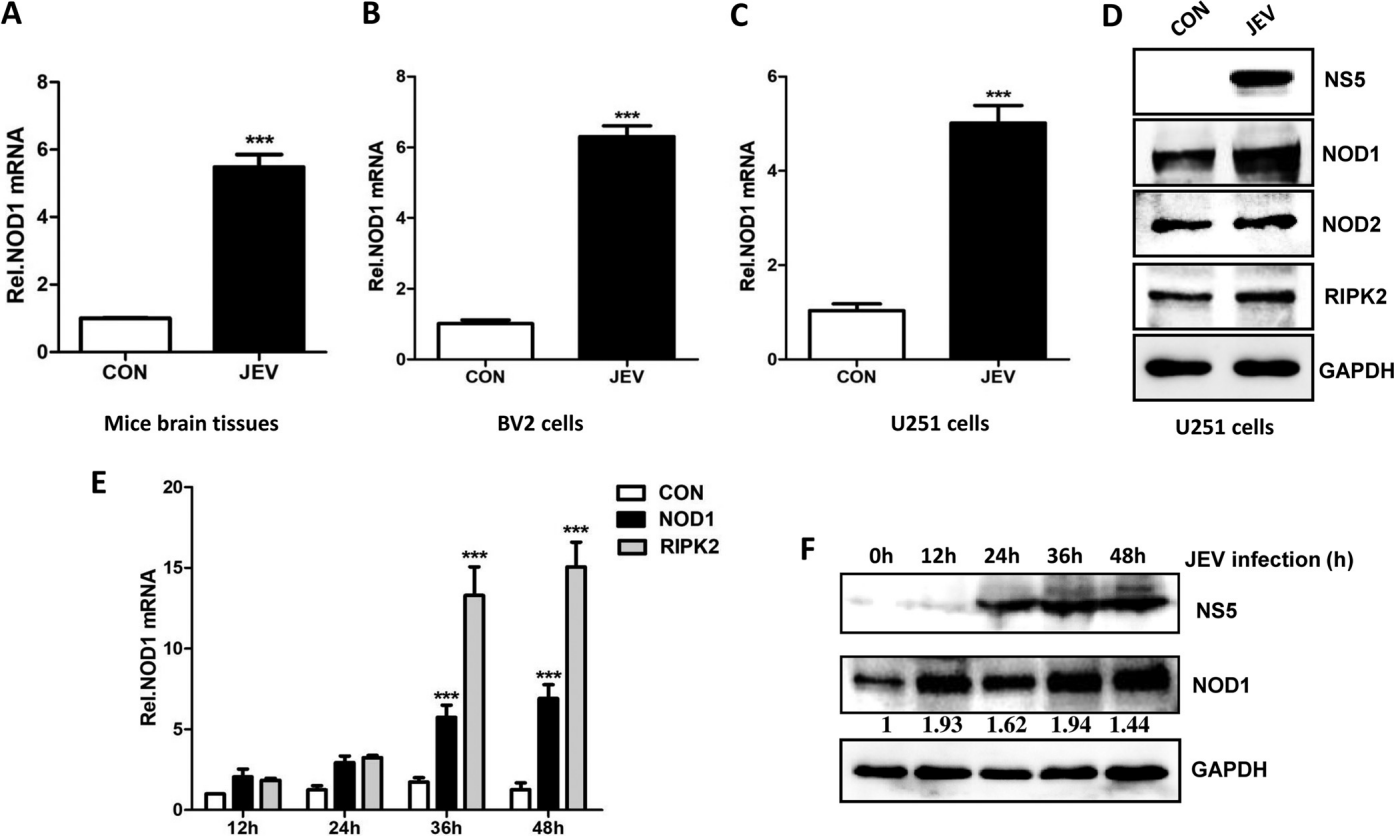
Japanese encephalitis virus (JEV) infection enhances the expressions of nucleotide-binding oligomerization domain 1 (NOD1) and receptor-interacting protein 2 (RIPK2) in mouse brain tissues and mouse or human glial cell lines. (A to C) Reverse transcription-quantitative PCR (qRT-PCR) analysis of NOD1 mRNA levels in (A) JEV-infected mice brain tissues, (B) BV2 cells, and (C) U251 cells. (D) Immunoblot analysis of NOD1, NOD2, and RIPK2 protein levels in U251 cells. (E) Quantitative real-time PCR (qRT-PCR) analysis of NOD1 and RIPK2 mRNA levels at different time points post-JEV infection of U251 cells. Cells were infected with JEV at an MOI of 5, followed by sample harvesting at indicated time points. (F) Immunoblot analysis of NOD1 protein levels at different time points post-JEV infection of U251 cells. Cells were infected with JEV at an MOI of 5, followed by sample harvesting at indicated time points. Data are presented as the results of three independent experiments. ***, *P* < 0.001.

The mechanism of JEV-induced neuroinflammation is very complex, because numerous host proteins are involved. Previously, we used a microarray approach to identify differentially expressed mRNAs in JEV-infected mouse brain tissues and found a large number of significantly expressed mRNAs that are predominantly involved in signaling pathways related to host immune and inflammatory responses (23). Among these, the expression of NOD1 mRNA was also observed to be significantly altered in infected brain tissues. To map the signaling pathways disturbed in mouse brains upon JEV infection, we assessed the effect of altered mRNA expression, as detected in our microarray study, on cell signaling pathways using the KEGG pathway and PathView bioinformatic algorithms. We found that the NOD1-RIPK2 signaling pathway was one of the pathways that were significantly perturbed after infection (Fig. S1A in the supplemental material). The mRNA expression levels of NOD1, RIPK2, and some other significantly expressed factors in brain tissues and mouse primary neuron/glia cultures were validated by quantitative real-time PCR (qRT-PCR) (Fig. S1B). Since the role of NOD1 in JEV infection is still unexplored, we focused on NOD1 for subsequent analyses. To further validate NOD1 and RIPK2 expression during JEV infection, we used newly prepared lysates of mouse brain tissues, mouse microglial cells (BV2), and human astrocytic cells (U251). NOD1 and RIPK2 showed upregulated expression levels upon JEV infection (Fig. 1A, B, and C), whereas a same-family member, NOD2, exhibited no change (Fig. 1D). Furthermore, we examined NOD1 and RIPK2 expression at different infection time points in cultured U251 cells and observed that JEV infection upregulated their expression in a time-dependent manner, as determined by qRT-PCR and immunoblot analysis (Fig. 1E and F). Overall, these results demonstrate that JEV infection enhanced the expression levels of NOD1 and RIPK2 *in vitro* and *in vivo*.

### NOD1 knockdown prevents JEV-induced inflammatory cytokine production *in vitro*.

**FIG 2:**
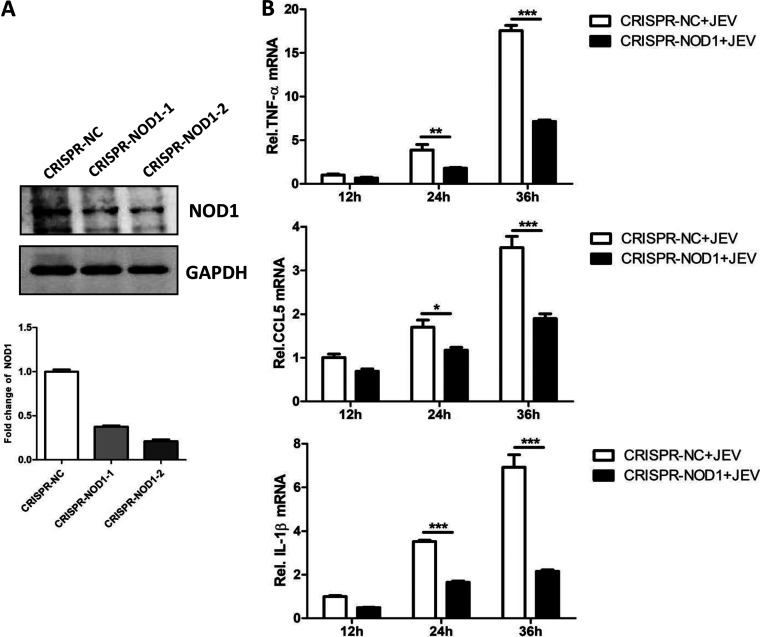
NOD1 knockdown inhibits JEV-induced inflammatory cytokine production *in vitro*. (A) Immunoblot analysis of NOD1 protein levels in U251 cells engineered for NOD1 knockdown using CRISPR-Cas9 technology. (B) qRT-PCR analysis of mRNA levels of inflammatory cytokines (tumor necrosis factor α [TNF-α], C-C motif chemokine ligand 5 [CCL5], and interleukin 1β [IL-1β]) at different time point post-JEV infection of U251 cells. Controls and NOD1 knockdown cells were infected with JEV at an MOI of 5, followed by sample harvesting at indicated time points. Data are presented as the results of three independent experiments. *, *P* < 0.05; **, *P* < 0.005; ***, *P* < 0.001.

Next, we investigated the role of NOD1 in regulating the JEV-induced inflammatory response. To explore this, we subjected U251 cells to NOD1 knockout using CRISPR/Cas9 technology. We remained unable to fully abrogate NOD1 expression, and hence, used cells displaying a NOD1 knockdown phenotype (Fig. 2A) for subsequent experiments. To this end, NOD1-engineered or control cells were infected with JEV at the indicated time points, followed by sample harvesting to determine the expressions of inflammatory cytokines. As shown in Fig. 2B, the knockdown of NOD1 significantly reduced the expression levels of tumor necrosis factor α (TNF-α), C-C motif chemokine ligand 5 (CCL5), and interleukin 1β (IL-1β) at 24 and 36 h postinfection compared to that in nonengineered cells. Thus, these data suggest a role of NOD1 in inducing the inflammatory response upon JEV infection.

### NOD1 knockout alleviates JEV-induced inflammatory response and glial cell activation in mouse brain tissues.

**FIG 3:**
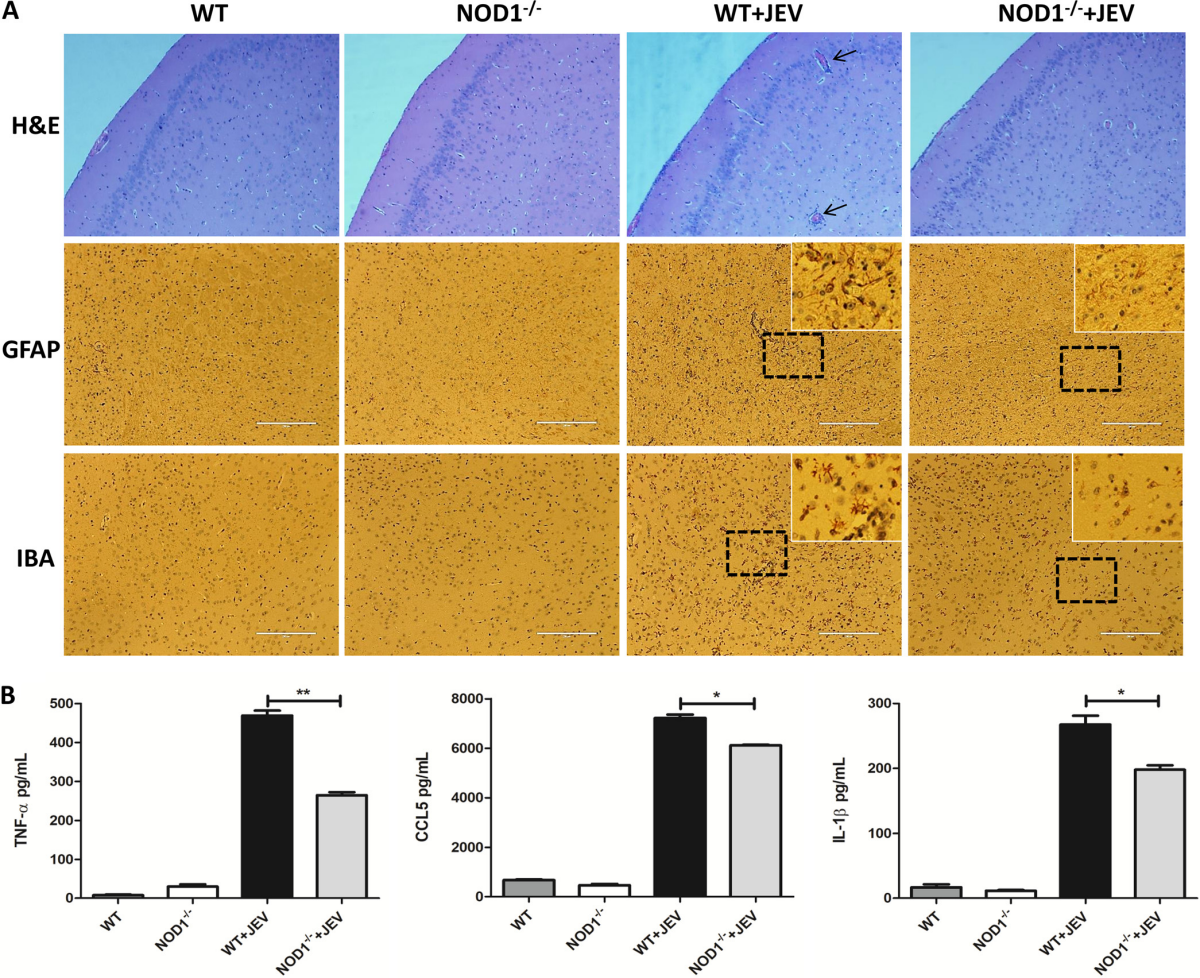
NOD1 knockdown alleviates JEV-induced inflammatory response and glial cell activation in mice brain tissues. (A) Hematoxylin and eosin (H&E) staining and immunohistochemistry (IHC) to observe pathological changes and activation of glial cells. Black arrows indicate perivascular cuffing and white boxes indicate activated glia cells. Scale bar, 200 μm. (B) WT mice and NOD1^–/–^ mice were subjected to JEV or mock infection as described previously. Brain samples were collected on day 7 after JEV infection. Protein levels of TNF-α, CCL-5, and IL-1β in brain lysates were analyzed by ELISA. Data are representative of three mice with similar results. *, *P* < 0.05, **, *P* < 0.01.

Inflammation is an important pathological manifestation of JE, and is due to the activation of glial cells and the secretion of inflammatory factors. To assess the role of NOD1 in JEV-induced neuroinflammation, wild-type (WT) or NOD1 knockout (NOD1^–/–^) mice were infected or not infected with JEV via the subperitoneal route. Mice from all groups were euthanized on day 7 postinfection when the WT mice exhibited obvious clinical symptoms of JE. Brain samples were collected and processed for inflammatory cytokine analysis, hematoxylin-eosin (H&E) staining, and immunohistochemistry (IHC). It was observed that the knockout of NOD1 expression in mice resulted in reduced histopathological changes (meningitis and perivascular cuffing) and reduced numbers of activated astrocytes and microglia in brain tissues compared to that in the WT mice (Fig. 3A). Moreover, the reduction of brain pathological features in NOD1^–/–^ mice was correlated with diminished secretion of inflammatory cytokines (TNF-α, CCL5, and IL-1β) in brain tissues (Fig. 3B). Taken together, these findings indicate that inhibition of NOD1 expression in mice reduces the neuroinflammation caused by JEV infection.

### NOD1 knockout reduces JEV-induced neuronal cell death in mouse brain tissues.

**FIG 4:**
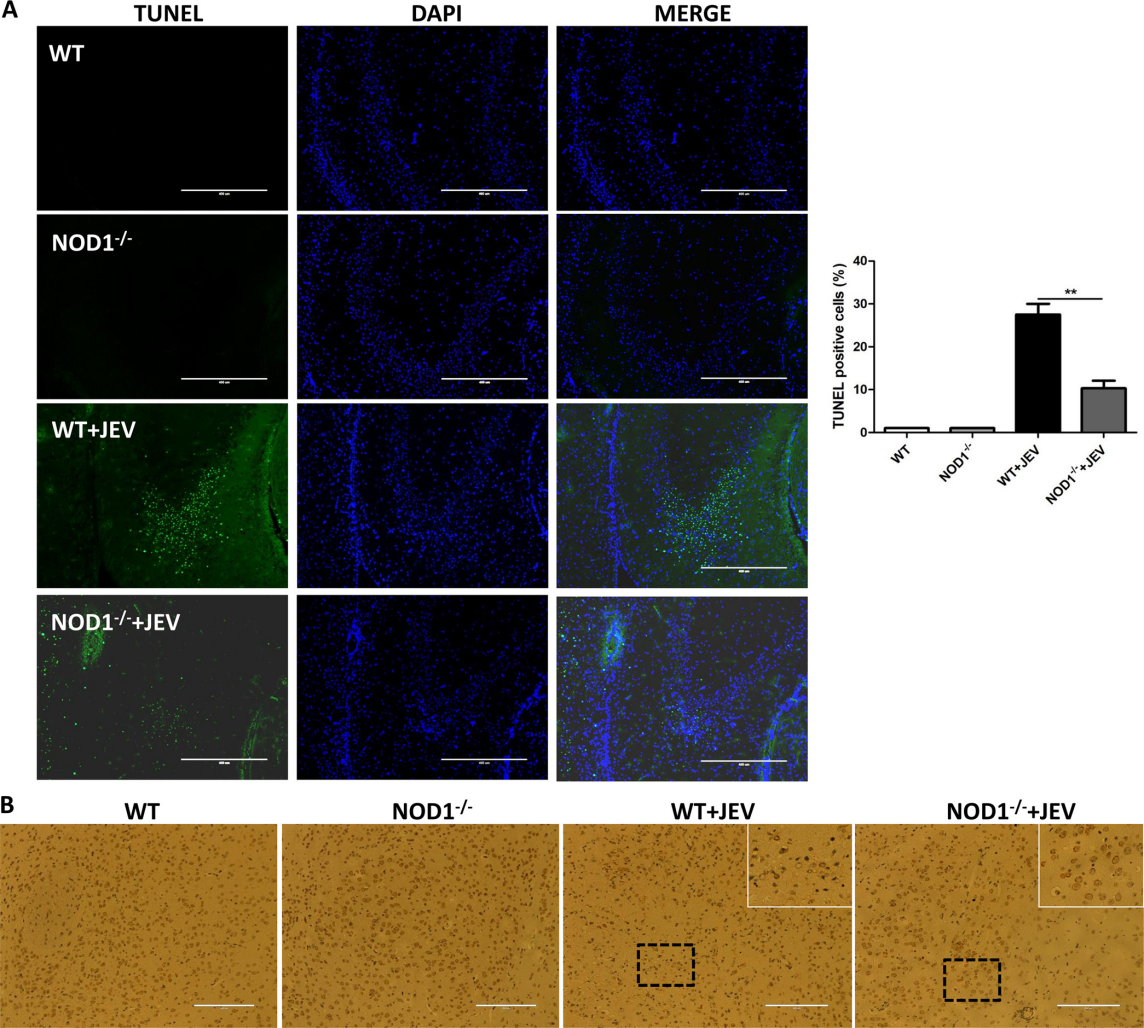
NOD1 knockout reduces JEV-induced neuronal cell death in mouse brain tissues. (A) Apoptotic neurons (green) in the mouse brain on day 7 after JEV infection were detected using a TUNEL (terminal deoxynucleotidyltransferase-mediated dUTP-biotin nick end labeling) assay kit. Right panel shows quantification of TUNEL-positive cells. Scale bar, 400 μm. (B) IHC staining to observe NeuN-positive cells. White boxes indicate neuronal degeneration and number changes. Scale bar, 200 μm. Data are representative of three mice with similar results. **, *P* < 0.01.

It has been reported that there is a close relationship between neuroinflammation and neuronal cell damage during JEV infection. Neuroinflammation can lead to neuronal cell damage, and inflammatory mediators released by dead neurons can also aggravate neuroinflammation (6, 8, 16, 24, 25). To examine whether NOD1 inhibition in mice can reduce neuronal cell damage, brain samples collected from JEV-infected or mock-infected WT or NOD1^–/–^ mice on day 7 postinfection were subjected to a terminal deoxynucleotidyltransferase-mediated dUTP-biotin nick end labeling (TUNEL) assay and IHC staining. The numbers of TUNEL-positive cells were found to be significantly decreased in JEV-infected NOD1^–/–^ mice compared to that in infected WT mice (Fig. 4A). Moreover, IHC staining showed a significant increase in the number of neuronal cells in JEV-infected NOD1 knockout mice compared to WT mice (Fig. 4B). Altogether, these data demonstrate that the inhibition of NOD1 in mice attenuates neuronal cell damage induced upon JEV infection.

### NOD1 knockout reduces mouse lethality induced by JEV infection.

**FIG 5:**
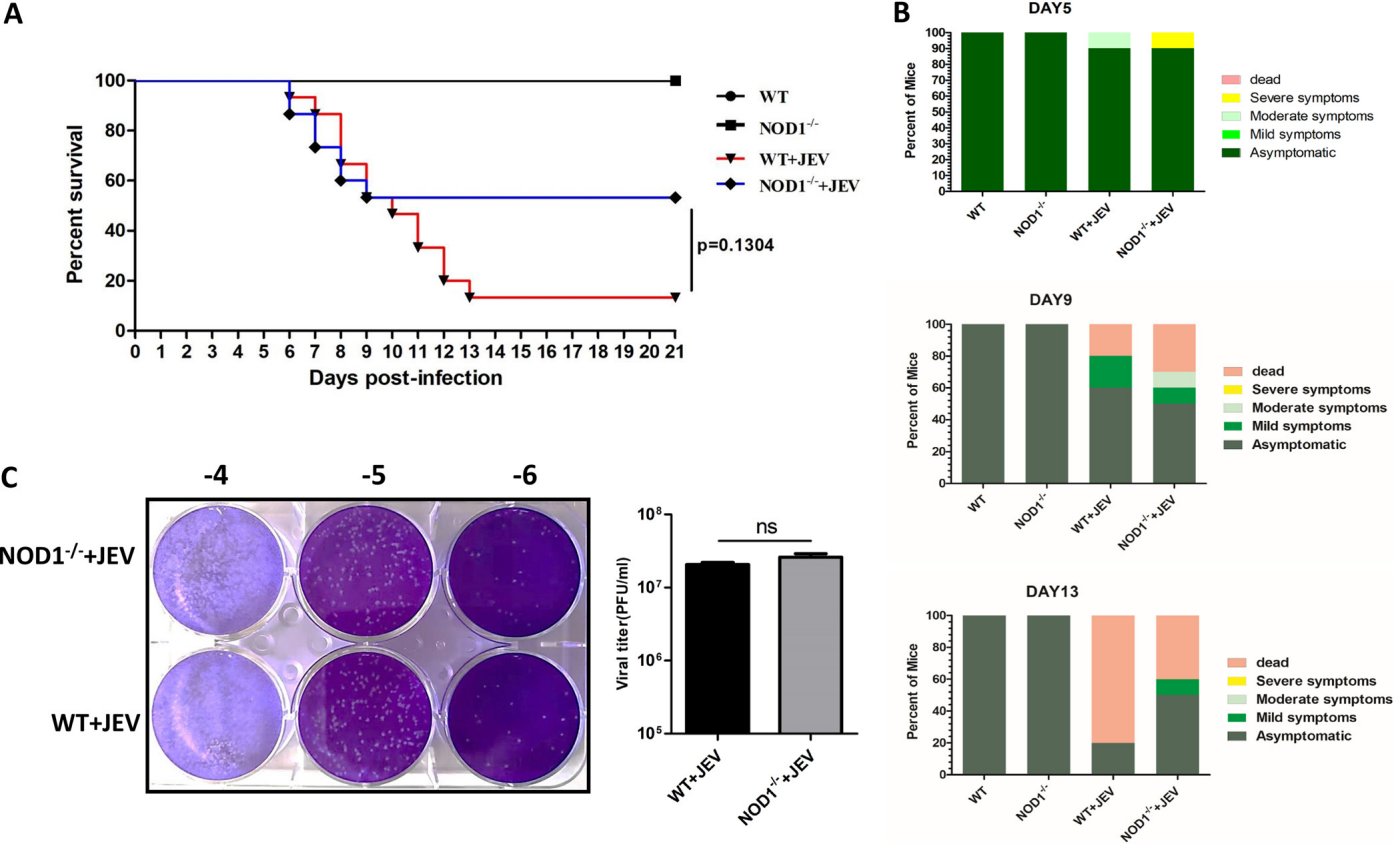
NOD1 knockout reduces lethality in JEV-infected mouse model. (A) Survival of mice in each group was monitored for 21 days after JEV inoculation. Data were collected and are shown as Kaplan-Meier survival curves (*n* = 15 mice). (B) Presentation of clinical signs of disease in mice on indicated days following JEV infection. (C) Viral titers in mouse brains on day 7 after JEV infection were determined by plaque assay. Viral titers are shown as PFU/mL (*n* = 5 mice).

As demonstrated above, NOD1 plays an imperative role in preventing the JEV-triggered inflammatory response and neuronal damage both *in vitro* and *in vivo*. We then assessed the potential of NOD1 knockout in protecting mice against JEV-associated lethality. In JEV-infected WT mice, mortality and morbidity were markedly higher than that in the infected NOD1^–/–^ mice. Mortality started to appear on day 6 postinfection and reached up to 86.7% on day 13. In contrast, the infected NOD1^–/–^ mice showed a lower mortality rate of 46.7% during the entire 21-day observation period (Fig. 5A). In addition, we recorded behavioral signs (such as messy hair, trembling, seizures, paralysis, and death) in all experimental groups of mice and found that the inhibition of NOD1 in infected mice caused improved behavioral signs compared to those in WT mice challenged with JEV infection (Fig. 5B). We also determined the viral loads in infected mouse brain tissues by measuring viral titers through plaque assay. Brain homogenates prepared on day 6 postinfection from JEV-infected NOD1^–/–^ mice showed almost equal viral titers compared to those from infected WT mice (Fig. 5C). These results suggest that the inhibition of NOD1 can provide a protective effect against JEV-induced mortality by dampening neuroinflammation.

### NOD1 regulates JEV-induced neuroinflammation through activation of the conventional targets: MAPK, ERK, JNK, and NF-κB.

**FIG 6:**
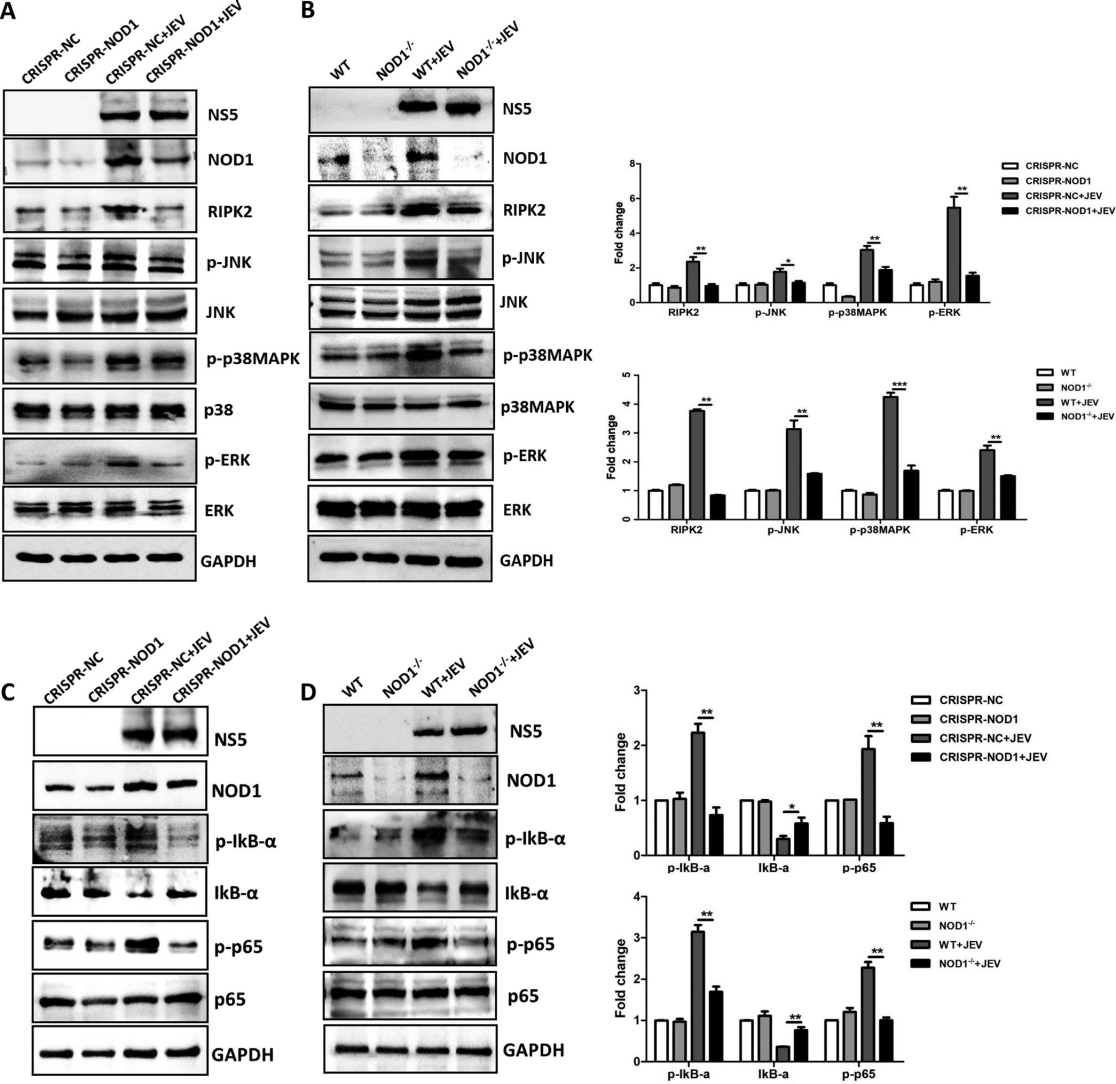
NOD1 signaling pathway regulates JEV-induced neuroinflammation. (A to D) Immunoblot analysis of expression or activation/phosphorylation of NOD1 and its associated downstream signaling molecules in NOD1 knockdown U251 cells and NOD1^–/–^ mouse brain tissues with or without JEV infection. Protein levels of RIPK2, p-JNK, p-p38MAPK, p-ERK, p-IκB-α, IκB-α and p-p65 were quantified by immunoblot scanning using image J software and normalized to the amount of GAPDH. Data are representative of three independent experiments or three mice with similar results. *, *P* < 0.05; **, *P* < 0.005; ***, *P* < 0.001.

The roles of NOD1-mediated signaling pathways in regulating inflammation have been studied extensively. The conventional downstream effector molecule for NOD1 is RIPK2 (26, 27), which initiates downstream signaling toward a variety of inflammation-related pathways such as MAPK, ERK, JNK activation, and NF-κB-dependent signaling (20, 21, 28–30). To determine the NOD1 signaling pathway involved in the regulation of JEV-induced neuroinflammation, we tested the activation of downstream molecules of NOD1 signaling in glial cells and mouse brain tissues upon JEV infection. As shown in Fig. 6A and B, increased NOD1 expression in WT U251 cells and mouse brain tissues was found to be associated with enhanced expression or activation/phosphorylation of its downstream signaling molecules (RIPK2, MAPK, ERK, and JNK), which ultimately led to NF-κB activation as confirmed by p65 activation and IκB degradation in the lysates of cultured cells and brain tissues. In contrast, the inhibition of NOD1 in cultured cells and mice markedly reduced the expression or activation/phosphorylation of NOD1 signaling-related molecules (Fig. 6A and B). Hence, these results suggest that NOD1 regulates JEV-induced neuroinflammation through activation of downstream MAPK, ERK, JNK, and NF-κB signaling pathways, both *in vitro* and *in vivo*.

### JEV infection enhances NOD1 expression by upregulating its transcription factor, IRF1.

**FIG 7:**
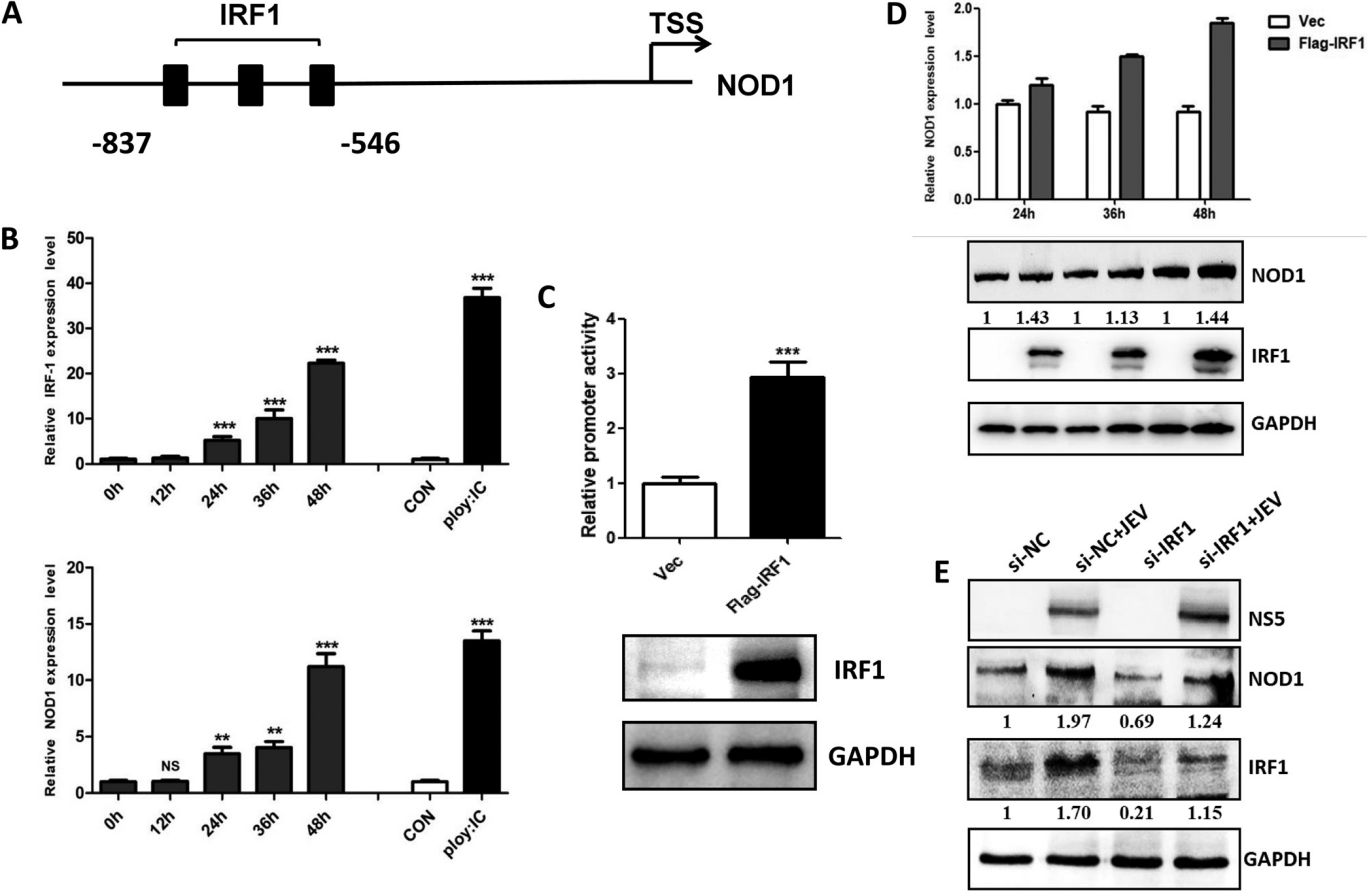
JEV infection upregulates NOD1 expression by enhancing the expression of transcription factor interferon regulatory factor 1 (IRF1). (A) Schematic diagram of the transcription factor IRF1 binding site upstream from the NOD1 promoter. (B) qRT-PCR analysis of IRF1 and NOD1 at different time points post-JEV infection and after poly: IC (polyinosinic-polycytidylic acid) treatment of U251 cells. (C) Analysis of NOD1 promoter activity in U251 cells using the dual-luciferase reporter assay system. Upper panel indicates luciferase signals, lower panel confirms Flag-tagged IRF1 expression in the transfected cells. (D) Transcriptional and post-transcriptional levels of NOD1 upon Flag-tagged IRF1 expression in U251 cells. (E) Immunoblot analysis of NOD1 protein levels after IRF1 small interfering RNA (siRNA) transfection followed by JEV infection of U251 cells. Cells were transfected with IRF1 siRNA for 24 h, then infected with JEV at an MOI of 5, followed by sample harvesting at indicated time points. Data are presented as the results of three independent experiments. **, *P* < 0.005; ***, *P* < 0.001.

NOD1 expression can be regulated at the transcriptional and post-transcriptional levels by various mechanisms. It is known that the interferon-γ (IFN-γ), a potent proinflammatory cytokine, can regulate the promoter activity of NOD1 by targeting its transcription factor, interferon regulatory factor 1 (IRF1) (31). There are three IRF1 binding sites in the core promoter region of NOD1, as shown in Fig. 7A. Since JEV infection is also known to augment the expression of interferon-γ, we hypothesized that JEV infection may promote NOD1 transcription by upregulating IRF1 expression. To confirm this, we first examined the expression level of IRF1 in JEV-infected U251 cells and found that JEV infection enhanced IRF1 expression levels in a time-dependent manner, which is analogous to NOD1 expression in these infected cells (Fig. 7B). In addition, we assessed NOD1 promoter activity in the presence or absence of IRF1 expression. As quantified by a luciferase reporter system, the activity of the NOD1 promoter region was significantly enhanced when Flag-tagged IRF1 was co-expressed in NOD1 promoter-expressing cells (Fig. 7C). IRF1-induced enhanced NOD1 promoter activity was also observed to associate with increased NOD1 expression at both the transcriptional and post-transcriptional levels, as determined by qRT-PCR and immunoblot analysis, respectively (Fig. 7D). In addition, IRF1 knockdown can significantly attenuate JEV-induced upregulation of NOD1 expression (Fig. 7E). Thus, these results reveal that JEV infection stimulates NOD1 expression by upregulating the expression of its transcription factor, IRF1.

## DISCUSSION

NOD1 and NOD2 are well-characterized intracellular PRRs which recognize γ-d-glutamyl-meso-diaminopimelic acid and muramyl dipeptide, respectively. γ-d-Glutamyl-meso-diaminopimelic acid is found predominantly in Gram-negative bacteria, while muramyl dipeptide is present in the peptidoglycans of almost all bacteria. NOD1 and NOD2 participate in regulating the innate immunity and inflammatory responses induced by bacteria (21, 32). In this study, we discovered for the first time that NOD1 positively regulates neuroinflammation caused by JEV infection.

Both NOD1 and NOD2 have broad tissue distribution. Expression of NOD1 and/or NOD2 has been documented in macrophages, myeloid dendritic cells (DCs), plasmacytoid DCs, B cells, CD4^+^ and CD8^+^ T cells, natural killer cells, γδ T cells, neutrophils, endothelial cells, adipocytes, smooth and skeletal muscle cells, and platelets. Each cell type appears to have different levels of NOD1 and NOD2 expression. Here, we detected high NOD1 expression in glial cells, especially in astrocytes, which are the targets of JEV in the CNS, preceded only by neurons, and proved the role of NOD1 in JEV pathogenesis. Whether or not NOD1 has the same functions in cells other than glial cells in the CNS remains undisclosed.

It has been documented that NOD1 and NOD2 exhibit similar structure and domain organization, but their functions are somewhat different (17, 19–21). In addition to differences in the recognition and regulation of bacterial infection, NOD1 and NOD2 also display different functions during viral infections. For instance, NOD1 can recognize dsRNA of the hepatitis C virus and subsequently trigger downstream RIG-I-mediated interferon-β (IFN-β) signaling (22), whereas NOD2 oligomerizes and interacts with the mitochondrial antiviral signaling protein to drive IRF3-dependent expression of IFN-β (33). In this study, we showed that JEV infection upregulated NOD1 expression, while inducing no effect on NOD2 expression. The increased NOD1 expression was found to enhance JEV pathogenesis by stimulating the conventional inflammatory pathways. Whether or not NOD2 participates during JEV infection remains undetermined.

NOD1 activation stimulates the NF-κB signaling pathway, leading to the production of IFN, which, in turn, inhibits virus replication (34). On the other hand, viruses have evolved strategies to exploit NF-κB signaling for the purpose of inducing inflammatory responses (35). These contradictory effects could be associated with tissue specificity. Considering this, we propose that NOD1 acts as an antiviral factor in the periphery, whereas in the CNS it acts as a proviral factor to enhance JEV pathogenesis. In addition, it has been reported that NOD1 can trigger IFN and inflammatory responses by engaging signaling pathways other than NF-κB (36–40). However, the exact mechanism for this needs to be explored.

Upon infection, NOD1 expression can be regulated by a variety of mechanisms. As mentioned previously, IFN-γ can regulate NOD1 promoter activity by targeting the transcription factor IRF1 to activate NOD1 mRNA transcription. In our study, we found that JEV infection enhanced IRF1 expression to activate the NOD1 promoter activity, thereby leading to increased NOD1 expression. Because the NOD1 promoter region harbors multiple transcription factor binding sites, it would be interesting to examine whether transcription factors other than IRF1 regulate NOD1 expression during JEV infection. Moreover, NOD1 was proven to be activated by the RNA-dependent RNA polymerase and dsRNA of the hepatitis C virus (21). In a similar context, we also found that the treatment of cultured cells with dsRNA artificial mimics (polyinosinic-polycytidylic acid, or poly I:C) promoted NOD1 expression at the transcription level (Fig. 7B), suggesting that there may be some unknown mechanisms which can also lead to the accumulation of NOD1; this needs to be explored in future studies.

In conclusion, we demonstrated for the first time that JEV infection activates the NOD1 signaling pathway to induce neuroinflammation. The inhibition of NOD1 reduces neuropathogenesis and protects mice from lethality during JEV infection. Therefore, targeting NOD1 could be considered a potential therapeutic approach to treat JEV pathogenesis.

## MATERIALS AND METHODS

### Cell culture and virus propagation.

Human astrocytoma cell line U251 and mouse microglial cell line BV-2 were cultured and maintained in Dulbecco’s modified Eagle’s medium (DMEM; 4,500 mg/L glucose) supplemented with 10% (vol/vol) heat-inactivated fetal bovine serum, penicillin (100 U/mL), and streptomycin sulfate (100 mg/mL) at 37°C in a 5% CO_2_ atmosphere. JEV wild-type strain P3 was propagated in suckling mouse brains and viral titers were determined by plaque assay on baby hamster kidney (BHK)-21 cells.

### NOD1 knockdown.

Two guide RNAs (gRNAs) targeting NOD1 and one control gRNA targeting green fluorescent protein were cloned into the lentiviral vector lentiCRISPR v2. About 800 ng lentiviral vector, 400 ng packaging plasmid pMD2.G, and 800 ng pSPAX2 was cotransfected into HEK 293T cells using the FuGENE HD transfection reagent (Promega, Madison, WI) according to the manufacturer’s instructions. At 48 h post-transfection, viral supernatants were collected and then inoculated into 4 × 10^5^ U251 cells for another 48 h. Next, the gRNA-expressing cells were selected with 1.5 μg/mL puromycin and then plated into 12-well plates for further experiments. The single-cell clones were cultured in 96-well plates for an additional 7 days or longer. Immunoblotting was used to screen for NOD1-deficient clones and verify the knockdown efficiency. The genotyping of the knockout cells was determined by sequencing. The sequences of the gRNAs were as follows: gRNA-NOD1-1, 5′-AAACCATCTTCGGCCGAGAAGTAGC-3′; gRNA-NOD1-2, 5′-AAACCGTAGGCATCTGCGAGTTGCC-3′.

### Preparation of primary mouse neuron/glia cultures.

Neuron/glia cultures were prepared from the cerebral cortices of embryonic day 15 (E15) C57BL6J wild-type mice and plated on polylysine-coated (20 mg/mL) dishes at a density of 10^5^ cells per well in DMEM supplemented with 5% fetal bovine serum. After 6 h of seeding, the culture medium was replaced with neurobasal medium supplemented with 2% B-27, 0.5% streptomycin and penicillin, and 0.5 mM l-Glu. These cells were used for subsequent experiments after incubation for 7 days. The neuron/glia cultures were mock-infected or infected with JEV at a multiplicity of infection (MOI) of 1, followed by sample harvesting at 36 h postinfection.

### Mouse experiments.

Adult 6-week-old WT and NOD1^–/–^ C57BL/6N mice were purchased from Cyagen Biotechnology (Santa Clara, CA). Mice were randomly divided into four groups: WT (*n* = 20), NOD1^–/–^ (*n* = 20), JEV-infected WT (JEV + WT; *n* = 20), and JEV-infected NOD1^–/–^ (JEV + NOD^–/–^; *n* = 20). Mice belonging to the JEV + WT and JEV + NOD1^–/–^ groups were intraperitoneally injected with 10^5^ PFU of JEV P3 strain in 200 μL DMEM. At 6 days postinfection, mice infected with JEV developed signs of viral encephalitis. Five mice from each group were euthanized and brain samples collected for further studies. The remaining 15 mice from each group were monitored daily to assess behavior and mortality. All animal experiments were performed following National Institutes of Health Guidelines for the Care and Use of Laboratory Animals, and the experimental protocols were approved by the Huazhong Agricultural University Research Ethics Committee of the College of Veterinary Medicine.

### Measurement of cytokine production.

Enzyme-linked immunosorbent assay (ELISA) kits (eBioscience Inc., San Diego, CA) were used to determine TNF-α, IL-1β, and CCL5 secretion in mouse brain tissue lysates, according to the procedure supplied by the manufacturer.

### RNA extraction and quantitative real-time PCR.

Total RNA was extracted using the TRIzol reagent (Invitrogen, Waltham, MA). About 1 μg of total RNA was used to synthesize the cDNA using a first-strand cDNA synthesis kit (ABclonal Technology, Woburn, MA). Quantitative real-time PCR was performed using a 7500 Real-Time PCR system (Applied Biosystems, Waltham, MA) and a SYBR Green PCR Master Mix (ABclonal Technology). The relative mRNA expression levels of TNF-α, CCL-5, and IL-1β were normalized to the mRNA levels of the endogenous control β-actin within each sample using the 2^−ΔΔCT^ (threshold cycle) method. The primers used were as follows: Hsa-IL-1β-F, 5′-GGCAATGAGGATGACTTGTTCT-3′; Hsa-IL-1β-R, 5′-CTGTAGTGGTGGTCGGAGATTC-3′; Hsa-CCL-5-F, 5′-CATATTCCTCGGACACCACAC-3′; Hsa-CCL-5-R, 5′-ATGTACTCCCGAACCCATTTC-3′; Hsa-TNF-α-F, 5′- GTTCCTCAGCCTCTTCTCCTTC-3′, Hsa-TNF-α-R, 5′- CTTGTCACTCGGGGTTCG -3′; Hsa-β-actin-F, 5′-AGCGGGAAATCGTGCGTGAC-3′; and Hsa-β-actin-R, 5′-GGAAGGAAGGCTGGAAGAGTG-3′.

### Immunoblotting.

Cell pellets or mouse brain tissues were lysed in radioimmunoprecipitation assay buffer (Sigma-Aldrich, St. Louis, MO) containing phosphatase (PhosSTOP; Roche AG, Basel, Switzerland) and protease inhibitors (Complete Tablets, Roche AG). Protein concentrations were measured using a BCA (bicinchoninic acid) Protein Assay kit (Thermo Fisher Scientific, Waltham, MA). Each sample was electrophoresed and transferred onto a polyvinylidene difluoride (PVDF) membrane. Membranes were then blocked by incubating in blocking buffer (Tris-buffered saline with 0.5% Tween 20 and 5% bovine serum albumin) for 1 h and probed with primary antibodies overnight at 4°C. After washing, membranes were incubated with peroxidase-conjugated secondary antibodies (ABclonal Technology). The blots were processed for development using the SuperSignal West Femto system (ABclonal Technology). The primary antibodies were used as follows: mouse monoclonal antibody against JEV NS5 (1 ng/mL for Western blot assay, prepared by our laboratory), rabbit polyclonal antibodies against NOD1 (Cell Signaling Technology, Danvers, MA), NOD2 (ABclonal Technology), RIPK2 (Cell Signaling Technology), NF-κB, IκB-α, JNK, p-JNK, p38MAPK, p-p38MAPK, ERK, p-ERK, and glyceraldehyde-3-phosphate dehydrogenase (GAPDH) (ABclonal Technology). Appropriate concentrations for these commercial antibodies were used, following the manufacturer’s guidelines.

### Hematoxylin-eosin staining, immunohistochemistry, and TUNEL assay.

Mice were anesthetized with ketamine-xylazine (0.1 mL per 10 g of body weight) and brain tissues were collected and embedded in paraffin for coronal sections. Standard H&E staining protocol was followed for tissue staining. For IHC staining, sections were incubated overnight at 4°C with primary antibodies against ionized calcium-binding adapter molecule 1 (Wako, Japan), glial fibrillary acidic protein (Dako, Denmark), and neuronal nuclei (NeuN; United Chemi-Con, Rolling Meadows, IL). After washing, slides were incubated with appropriate secondary antibodies, washed again, and cover-protected. The TUNEL assay was performed using the In Situ Cell Death Detection Kit (Roche), following the manufacturer’s instructions.

### Titration of virus.

Virus loads in culture supernatants and mouse brain tissues were assessed by plaque assay on BHK-21 cells, as described previously (25). Results were measured as PFU per g of tissue weight or mL of supernatant.

### Statistical analysis.

All experiments were carried out at least three times under similar conditions. Analyses were conducted using Prism5 software (GraphPad). Results are shown as the mean ± standard error of the mean (SEM) except for viral loads, which are expressed as the median. Statistical differences between the experimental groups were determined using the Student’s *t* test. *P* values of  <0.05 were considered significant.
