# Supplementary material for: Nucleotide-Binding Oligomerization Domain 1 (NOD1) Positively Regulates Neuroinflammation during Japanese Encephalitis Virus Infection
Source: Microbiol Spectr. 2022 May 31;10(3):e02583-21. doi: 10.1128/spectrum.02583-21 (PMC9241932; doi:10.1128/spectrum.02583-21)
Supplement: SUPPLEMENTAL FILE 1 — Supplemental material. Download spectrum.02583-21-s001.pdf, PDF file, 0.6 MB [file spectrum.02583-21-s001.pdf]

A

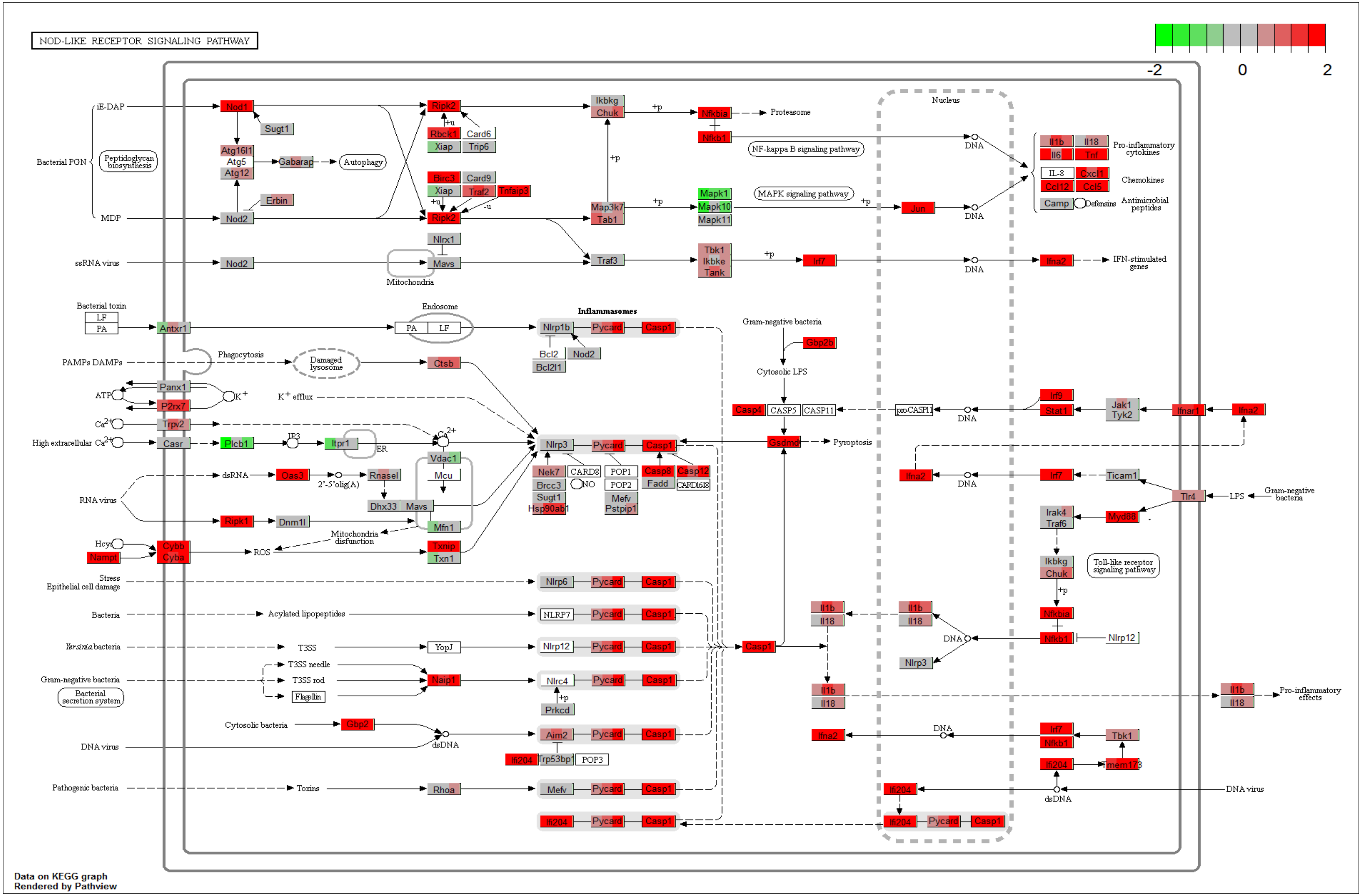
NOD-LIKE RECEPTOR SIGNALING PATHWAY
-2
0
2
Nucleus
iE-DAP
Bacterial PGN
Peptidoglycan biosynthesis
MDP
Nod1
Sugt1
Atg16l1
Atg5
Atg12
Gabarap
Autophagy
Erbin
Nod2
Ripk2
Rbck1
Card6
Xiap
Trip6
Birc3
Card9
Traf2
Tnfaip3
Ikbkg
Chuk
Nfkbia
Proteasome
Nfkb1
NF-kappa B signaling pathway
DNA
Map3k7
Tab1
Mapk1
Mapk10
Mapk11
MAPK signaling pathway
Jun
Il1b
Il18
Il6
Tnf
Pro-inflammatory cytokines
IL-8
Cxcl1
Ccl12
Ccl5
Chemokines
Camp
Defensins
Antimicrobial peptides
ssRNA virus
Nlrx1
Mavs
Mitochondria
Traf3
Tbk1
Ikbke
Tank
Irf7
Ifna2
IFN-stimulated genes
Bacterial toxin
LF
PA
Antxr1
Endosome
Inflammasomes
Nlrp1b
Pycard
Casp1
Bcl2
Bcl2l1
Gram-negative bacteria
Gbp2b
Cytosolic LPS
PAMPs DAMPs
Phagocytosis
Damaged lysosome
Ctsb
Panx1
ATP
K+
K+ efflux
P2rx7
Casp4
CASP5
CASP11
proCASP1
Irf9
Stat1
Jak1
Tyk2
Ifnar1
Ca2+
Trpv2
High extracellular Ca2+
Casr
Plcb1
IP3
Itpr1
ER
Nlrp3
Gsdmd
Pyroptosis
Vdac1
Mcu
Nek7
CARD8
POP1
Casp8
Casp12
Brcc3
NO
POP2
Fadd
CARD16/18
Sugt1
Mefv
Hsp90ab1
Pstpip1
dsRNA
Oas3
2'-5'olig(A)
Rnasel
RNA virus
Dhx33
Ticam1
Tlr4
LPS
Gram-negative bacteria
Ripk1
Dnm1l
Mfn1
Irak4
Traf6
Myd88
Mitochondria disfunction
Hcys
Cybb
Cyba
Nampt
ROS
Txnip
Txn1
Toll-like receptor signaling pathway
Stress
Epithelial cell damage
Nlrp6
Bacteria
Acylated lipopeptides
NLRP7
Yersinia bacteria
T3SS
YopJ
Nlrp12
T3SS needle
T3SS rod
Flagellin
Gram-negative bacteria
Bacterial secretion system
Naip1
Nlrc4
Prkcd
Cytosolic bacteria
Gbp2
dsDNA
DNA virus
Aim2
Ifi204
Trp53bp1
POP3
Pathogenic bacteria
Toxins
Rhoa
Nlrp3
Pro-inflammatory effects
Tmem173
DNA virus
Data on KEGG graph
Rendered by Pathview

B

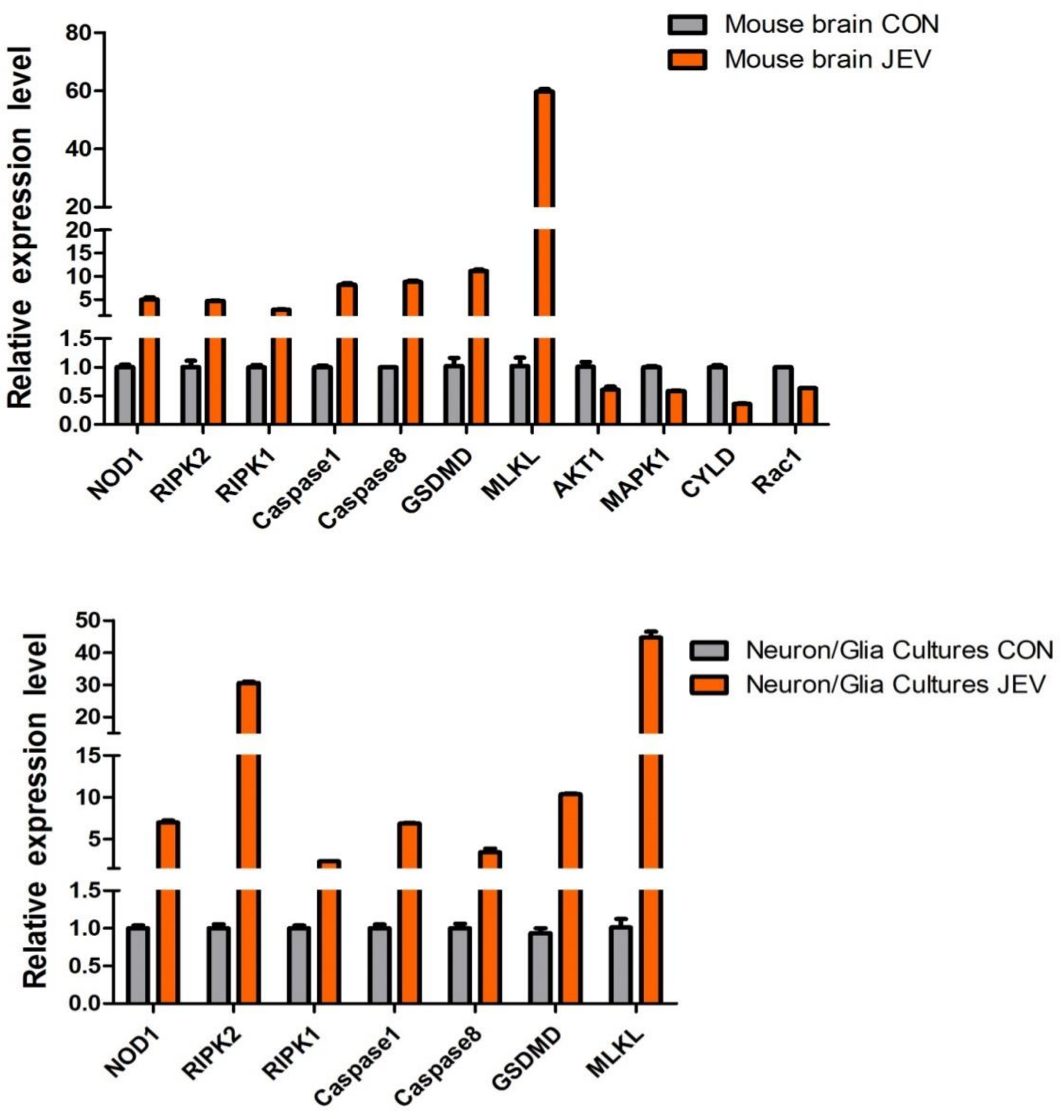
Mouse brain CON
Mouse brain JEV
Relative expression level
80
60
40
20
20
15
10
5
1.5
1.0
0.5
0.0
NOD1
RIPK2
RIPK1
Caspase1
Caspase8
GSDMD
MLKL
AKT1
MAPK1
CYLD
Rac1
Neuron/Glia Cultures CON
Neuron/Glia Cultures JEV
Relative expression level
50
40
30
20
15
10
5
1.5
1.0
0.5
0.0
NOD1
RIPK2
RIPK1
Caspase1
Caspase8
GSDMD
MLKL

FIG S1. (A) Visualization of NOD1-RIPK2 signal in KEGG pathways with significant perturbation by PathView (Red and green represent up and downregulated expressions, respectively). (B) qRT-PCR validation of gene expressions in JEV-infected mice brain tissues and mouse primary neuron/glia cultures. Data are presented as the results of three independent experiments.
